# Ingesting chitosan can promote excretion of microplastics

**DOI:** 10.1038/s41598-025-96393-w

**Published:** 2025-04-23

**Authors:** Di Liu, Muneshige Shimizu

**Affiliations:** 1 Graduate School of Science and Technology, Tokai University, 3-20-1 Orido, Shimizu, 424- 8610 Shizuoka Japan; 2 Institute Oceanic Research and Development, Tokai University, 3-20-1 Orido, Shimizu, 424- 8610 Shizuoka Japan

**Keywords:** Microplastic, Excretion, Metabolism, Chitosan, Animal model, Microplastic mitigation, Nutrition, Nutritional supplements

## Abstract

Recently, the presence of microplastics (MPs) in common foods such as salt and beverages has been widely reported Microplastics (MPs) have been widely reported in common foods, including salt and beverages. MPs spread through the food chain and are eventually ingested into the human body through the diet. They have been found to accumulate in human feces, blood, and liver tissues, raising concerns about the effects of continuous intake of foods containing MP on the body. We examined whether rats could rapidly excrete polyethylene MPs (average particle size of 200 μm) when the MPs and were mixed with non-digestive dietary materials in their feed (indigestible dextrin, lactosucrose, chitosan, and eggshell membrane proteins). The group that ingested chitosan showed significant changes, including increased fecal weight, increased MP excretion rate, and decreased intestinal MP retention rate. The MP excretion rates in feces 0–144 h after ingestion were 83.7% ± 3.8% in the control group and 115.6% ± 4.5% in the chitosan group. These findings indicate that chitosan effectively promotes the expulsion of polyethylene MPs. The addition of chitosan to food may reduce the potential harm caused by MPa to a variety of organisms, including humans.

**Supplementary Information:**

The online version contains supplementary material available at 10.1038/s41598-025-96393-w.

## Introduction

Microplastics (MPs) are minute plastics with a diameter of 5 mm or less; they can be classified as primary or secondary. Primary MPs, such as those used as scrubbing agents in facial cleansers and toothpaste, enter the ocean through household drains and sewage treatment systems^1–3^. However, secondary MPs are formed when larger plastic items, such as plastic bags, polyethylene terephthalate bottles, and cigarette filters, break down into smaller particles because of factors such as ultraviolet light and wave action. Secondary MPs are produced in large quantities in the ocean, raising concerns regarding their impact on marine organisms^4–9^. As a result, seafood and processed seafood products are more susceptible to the influence of MPs present in seawater than are agricultural products, leading to numerous reports on the presence of MPs in fish and canned seafood^10–16^. In addition, studies have identified MPs of < 50 μm in various human food products, including table salt, beer, and mineral water^17–25^.

Studies on the effects of MPs in living organisms, including humans, have shown the accumulation of MPs in various tissues, along with alterations in blood cholesterol and triglyceride levels in mice^26–29^. In addition, MPs have been identified in human feces, heart, and brain, and have been linked to increasing incidence of cardiac hypertrophy and atherosclerosis^14,30–33^. Accumulation of MPs in the gastrointestinal tract cause changes in the intestinal flora and production of inflammatory cytokines. Moreover, MP absorption into the body through the intestinal tract increases the risk of myocardial infarction, stroke, and death in animals and humans. Therefore, rapid excretion of MPs via the digestive tract and breathing is critical for maintaining the health of living organisms.

Non-digestive food materials are those that are not easily broken down by humangastrointestinal enzymes, such as dietary fiber. A wide variety of such materials exist and have diverse functional properties. Indigestible dextrin derived from corn is a water-soluble dietary fiber used in various health foods, with reported physiological effects such as the inhibition of postprandial triglycerides and blood glucose elevation^34^. Chitosan is an insoluble dietary fiber produced by deacetylation after removing proteins and calcium from chitin made from shrimp and crab shells; a significant reduction in total cholesterol and triglyceride levels has been reported in humans with continued intake of chitosan^35,36^. Lactosucrose is primarily made from lactose and sucrose and has been reported to suppress elevated postprandial blood glucose levels and improve the intentional environment^37,38^. Eggshell membrane proteins are non-digestive proteins that reportedly improve intestinal flora, enhance lipid metabolism, and inhibit visceral fat accumulation^39^.

When MPs are orally ingested by organisms, including humans, the above-mentioned offer substances offer the potential to rapidly excrete MPs as feces. However, to the best of our knowledge, no studies on this topic have been published. To address this issue, we conducted a basic study using rats to clarify whether ingesting non-digestive dietary materials contributes to the rapid excretion of MPs. To comprehensively evaluate the impact of various indigestible substances on microplastic excretion, we utilized distinct indigestible ingredients across different experimental groups. Each material possesses unique physical and chemical properties that may influence the excretion mechanism of microplastics. Although it has been reported that activated carbon can reduce MP contamination in water^40^, no studies have been conducted on food materials that are safe for human consumption. Through this experiment, we were able to comprehensively understand the roles of different components in the excretion of MPs, providing an important reference for future research.

## Methods

### Animals and experimental diet

We obtained 9-week-old Sprague–Dawley rats from the SLC Corporation of Japan; the rats were housed individually in a cage under controlled conditions: temperature (23℃ ± 1℃), humidity (35% ± 5%), and a 12 h light/dark cycle (light on, 8:00–20:00). After 1 week of adaptation, rats were divided into five groups (*n* = 6) based on uniformity of body weight and fed their respective diets for 1 week.

The diet was based on the AIN-93 M composition published by the U.S. National Institute of Nutrition, which is a standard rodent diet for laboratory animals. After acclimation to a basic diet consisting of AIN-93M^41^ for 1 week, 30 Sprague–Dawley rats were divided into five groups based on body weight—control group (Group C), indigestible dextrin group (Group D), lactosucrose group (Group O), chitosan group (Group K), and eggshell membrane group (Group E)—and kept in a metabolism cage for 1 week. A control diet was prepared by adding 0.72 g of polyethylene (PE) particles (Cospheric Inc., BLPMS-1.00-180 ~ 212 μm, 2.54 × 10^5^ particles/g) with an average particle size of 200 μm to 1 kg of a basic diet with AIN-93 M composition (Table 1). This is a common and easily bioabsorbable particle size in the environment. We chose specific colors (bule) and shapes (spherical) of MPs to facilitate observation and tracking, ensuring the reliability of the experimental results. In Group E, 5% of the protein content in the control diet was replaced with eggshell membrane protein. Groups D, O, and K were fed diets in which 5% of the corn starch in the control diet was replaced with each respective material (Table 1).

**Table 1:**
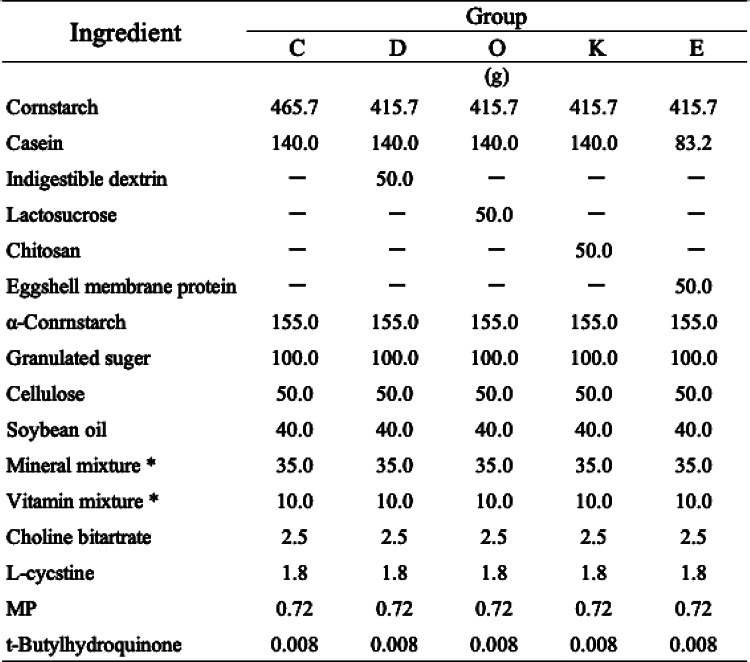
Compositions of experimental diets. *Mineral and vitamin mixtures were prepared according to AIN-93 M.

Throughout the feeding period, rats were free to eat and drink. A fresh diet and Milli-Q water were provided daily. Body weight, food intake, water consumption, and fecal weight were recorded daily. Fecal material was collected and stored at − 80 °C. After feeding, the rats were anesthetized with isoflurane and dissected. Blood samples were extracted from the abdominal aorta and centrifuged at 4℃ and 1,900 ×g for 10 min to obtain plasma. The liver, heart, kidneys, gastrointestinal tract, and fat (epididymal or perirenal fat) were dissected, weighed, and stored in liquid nitrogen. All samples were stored at − 80 °C before analysis. All experiments complied with the Animal Research: Reporting of In Vivo Experiments (ARRIVE) guidelines and were performed in compliance with the relevant Japanese and institutional laws and guidelines. The protocol was approved by the Committee on the Ethics of Animal Experiments of Tokai University (approval number 201085).

### Plasma biochemical analyses

To evaluate lipid metabolism in rat plasma, we examined changes in plasma total cholesterol and triglyceride levels after exposure to MPs. These markers were detected using a commercial kit (Wako Pure Pharmaceutical Co., Ltd., Osaka, Japan) and measurements were conducted according to the manufacturer’s protocol.

### Measurement of MPs in fecal matter

Fecal samples stored at − 80 °C were transferred to a freezer at − 25 °C for 12 h, then allowed to thaw naturally before being homogenized. Subsequently, 40 mg of the homogenized sample was accurately weighed using an electronic balance (ER-180 A; A&D Co., Ltd., Tokyo, Japan). The homogenized sample was treated with 10% KOH solution and stirred at 50 °C for 24 h; MPs were collected by suction filtration through a polytetrafluoroethylene (PTFE) membrane (OmniporeTM 5 μm × φ47 mm)^42–46^. Thereafter, the membrane was dried overnight at room temperature on a clean bench, and the number of MPs was counted using a LCD digital microscope DIM-03 (Alfa Mirage, Co. Ltd.).

### Measurement of MPs in the Gastrointestinal tract

The gastrointestinal tract, which had been stored at − 80 °C, was transferred to a − 25 °C freezer for 12 h and allowed to thaw naturally. The stomach, small intestine, cecum, colon, and rectum were excised. The contents of the stomach and cecum were collected using a spatula and washed with a 10% KOH solution. The collected contents of the small intestine, colon, and rectum were treated with 10% KOH. The five samples were stirred at 50 °C for 24 h, and the MPs were collected by suction filtration on a PTFE membrane (OmniporeTM 5 μm × φ47 mm). Thereafter, the membrane was dried overnight at room temperature on a clean bench, and the number of MPs was counted using a LCD digital microscope DIM-03 (Alfa Mirage, Co. Ltd.).

### Statistical analysis

Data are presented as the mean ± standard error (SE). For comparisons between groups, we performed single-placement variance analysis and multiple comparisons using Dunnett’s method. EZR (Easy R) was used for the analysis. A significance test for each data point was performed at a significance level of 5%.

## Results

### Body weight and food intake

**Fig. 1:**
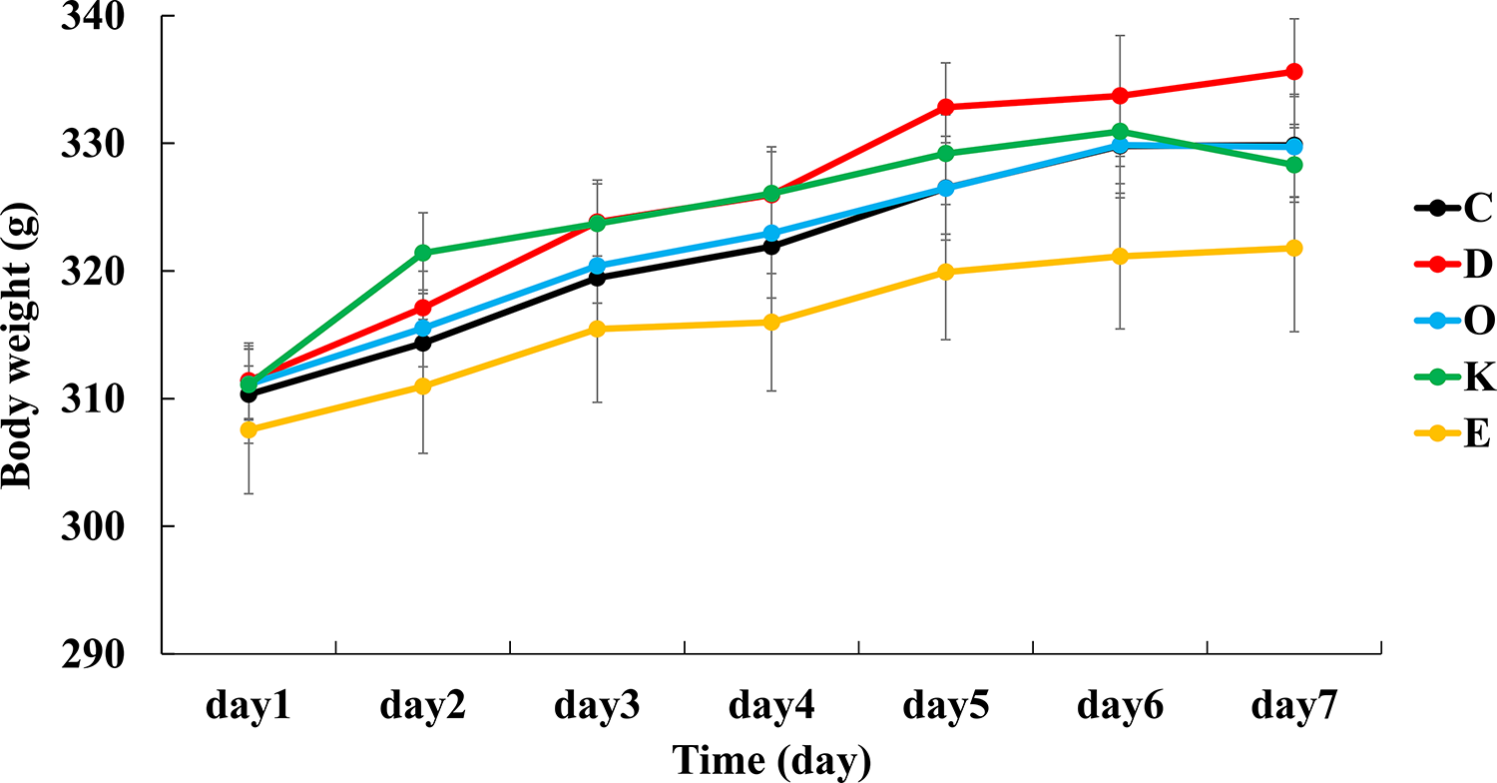
Body weight. Changes in body weight during the test period. No significant differences were observed among the groups. Values are the mean ± SE for six rats per group.

Following the addition of MPs to the diet, body weight continued to increase steadily. All groups showed similar increases after 1 week, with no significant differences observed among the groups (Fig. 1). The average daily food intake ranged from 17.5 to 17.8 g, and there were no significant differences among the groups.

### Fecal weight and tissue weight at dissection

**Fig. 2:**
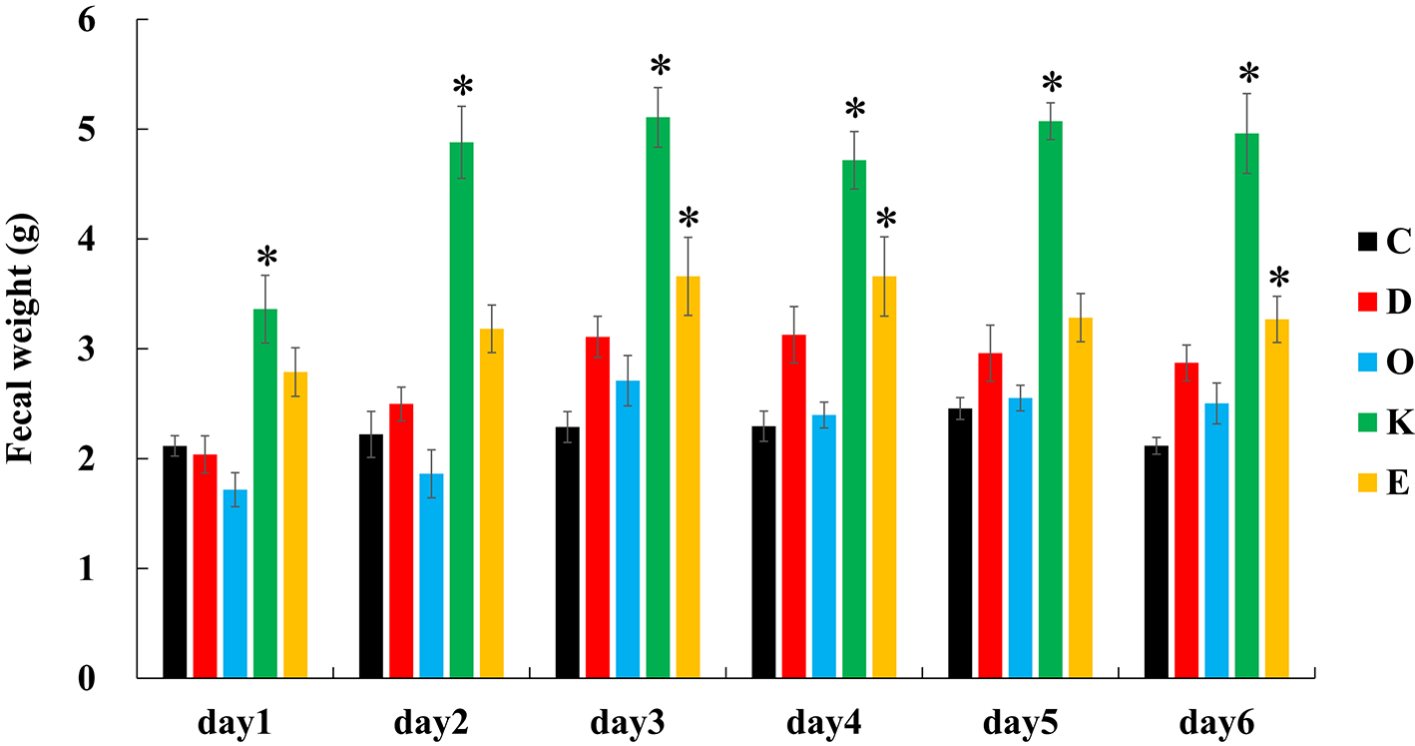
Fecal weight. Diurnal changes were observed from the start of the MP diet until the 6th day (days 1–6). Values are the mean ± SE for six rats per group. *p < 0.05, represents a significant difference compared with the control group (Group C).

Figure 2 shows the change in fecal weight from days 1 to6. From day 1 onwards, Group K exhibited a significant increase compared with that in Group C. Additionally, from day 2 onwards, Group E showed a significant increase compared with that in Group C. In contrast, the value in Group D was higher than that in Group C, but there was no significant difference (day 1, *p* = 1.00; day 2, *p* = 0.83; day 3, *p* = 0.14; day 4, *p* = 0.15; day 5, *p* = 0.32; and day 6, *p* = 0.09). Group O showed similar trends to those of Group C, with no significant differences observed among the groups.

The weight of each tissue per 100 g of body weight at the time of dissection is shown in Table 2. In the gastrointestinal tract, groups D and K showed significantly higher values than did Group C. No significant differences were observed among the groups in the other tissues.

**Table 2:**
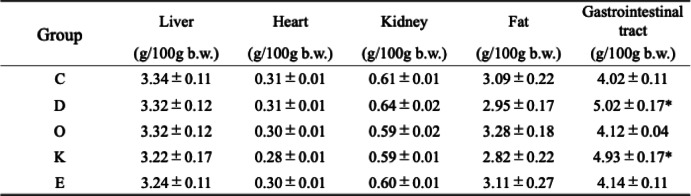
Tissue weight at dissection. Values are the mean ± se for six rats per group. ‘Fat’ denotes perirenal fat and epididymal fat. *p < 0.05 represents a significant difference compared with the control group (Group C).

### Plasma lipid concentrations

The changes in plasma lipid concentrations are shown in Table 3. During the 1-week experiment, no significant differences were observed in the plasma T-cho and TG levels among the groups.

**Table 3:**
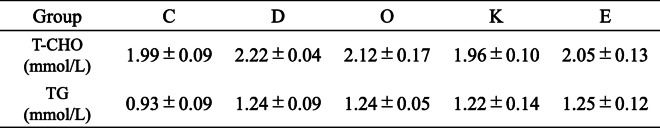
Total cholesterol and triglycerides in plasma. Values are the mean ± se for six rats per group. No significant differences were observed among the groups.

### MP excretion rate in fecal

**Fig. 3:**
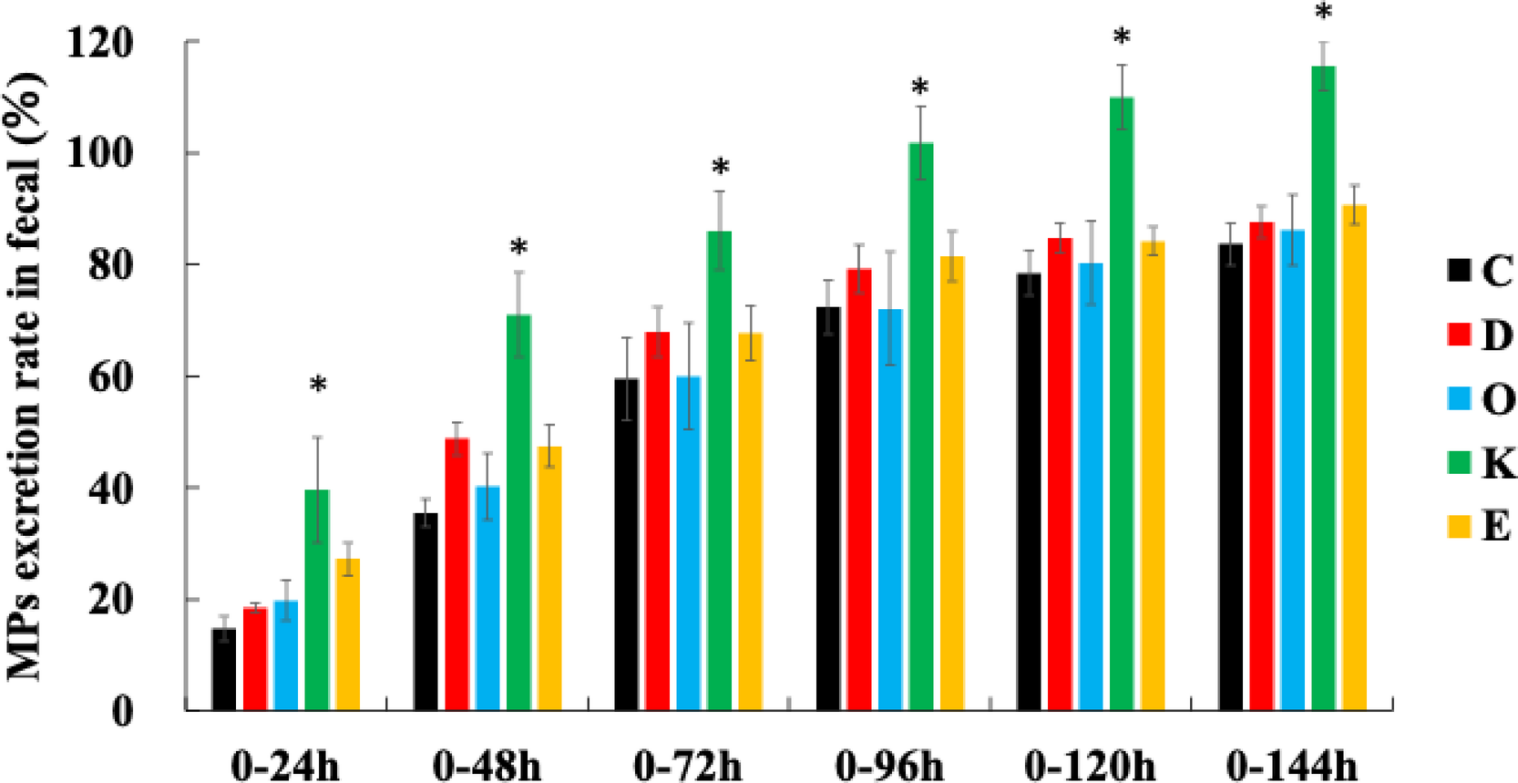
MP excretion rate in fecal matter. The MP excretion rate was calculated as the ratio of the number of MPs ingested from food during the given time period to the number of MPs excreted in feces. Values are the mean ± SE for six rats per group. *p < 0.05, represents a significant difference compared with the control group (Group C).

Figure 3 shows the fecal excretion rate of MPs from the start of the MP diet until Day 7 (0–144 h). In Group C, the fecal excretion rates were as follows: 0–24 h, 14.8% ± 2.2%; 0–144 h: 83.7% ± 3.8%. Group K showed significantly higher rates throughout all periods (0–24 h: 39.6% ± 9.4%, 0–144 h: 115.6% ± 4.5%). Groups D, O, and E were not significantly different from Group C at any time (Group D 0–48 h: *p* = 0.27, Group E 0–48 h: *p* = 0.36). (The specific data are presented in Supplementary Table 3.)

### MP excretion rate in the Gastrointestinal tract

**Fig. 4:**
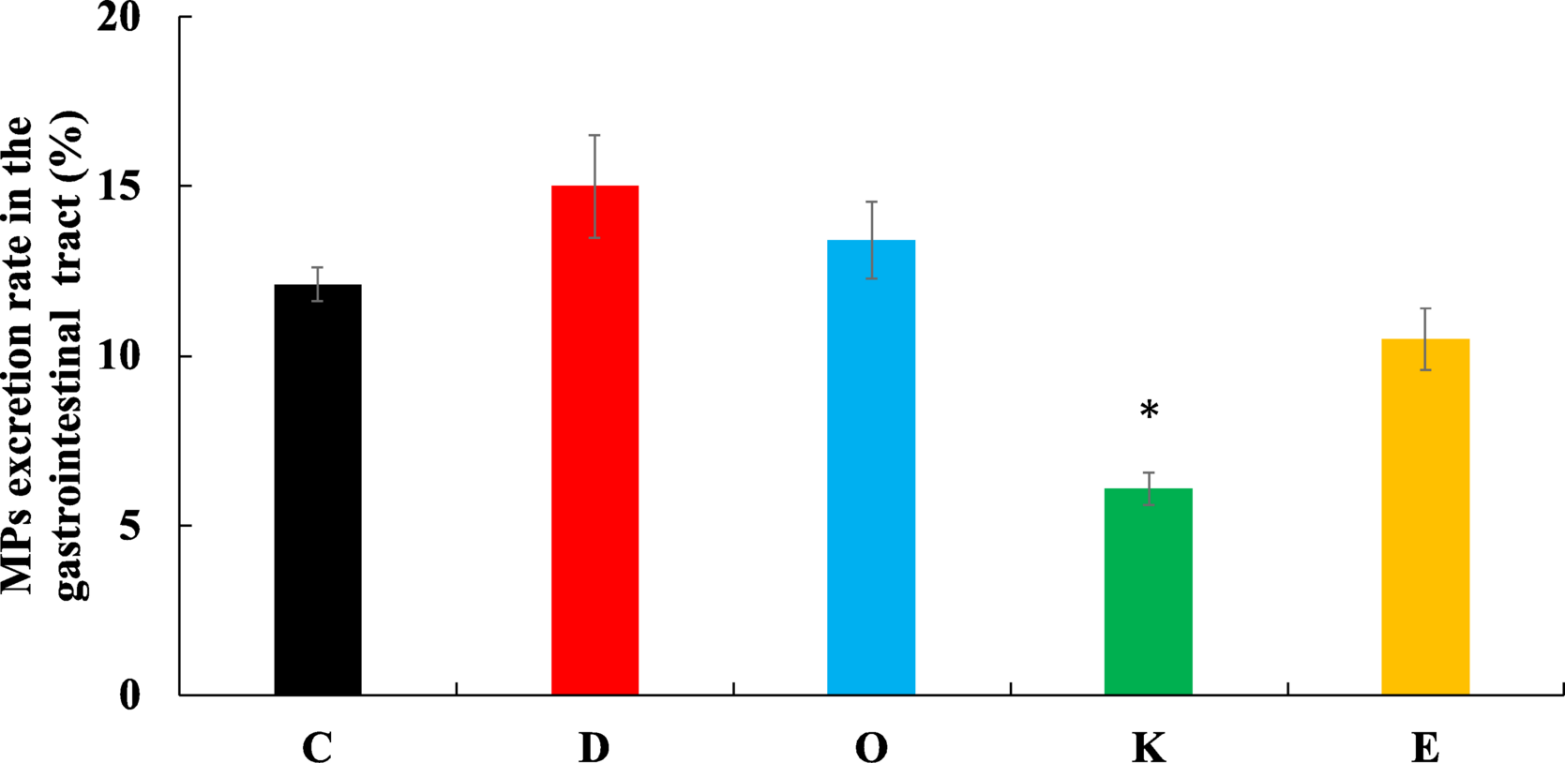
MP excretion rate in the gastrointestinal tract. Values are the mean ± SE for six rats per group. *p < 0.05 represents a significant difference compared with the control group (Group C).

Figure 4 shows the gastrointestinal excretion rate of MPs at the time of dissection. Group K (6.1% ± 0.5%) demonstrated a significantly lower rate compared with that of Group C (12.1% ± 0.5%). Groups D, O, and E were not significantly different from Group C. (The specific data are presented in Supplementary Table 4.)

## Discussion

In this study, we observed for the first time that orally ingested MPs remained in the gastrointestinal tract, mainly in the cecum, for a short period of 1 week. Furthermore, we clarified that, among the non-digestive materials tested, chitosan can rapidly excrete MPs.

We used the standardized PE particles (average particle size: 200 μm, number of particles: 2.54 × 10^5^ particles/g) for a purified diet (MP intake: approximately 3300 particles/day/rat). The body weight changes in groups D, O, K, and E were similar to those in Group C. Park et al. reported that when male ICR mice were continuously fed MPs with a particle size of approximately 20 μm for 90 days (4.3 × 10^5^ particles/day/mouse), there was no difference in body weight compared with that in the non-MP-fed group^47^. Compared with previous reports, MP intake in this study was lower, and the duration of MP intake was shorter; therefore, these results seem to be rescuable.

Fukuda et al. reported that by feeding SD rats purified feed AIN-93 M for 2 weeks (20.5 g/day/rat), the average fecal weight per rat was 1.33 g/day^48^. Saito et al. fed purified SD rats AIN-76 for 4 weeks (19.2 g/day/rat), resulting in an average fecal weight of 1.87 g/day per rat^49^. In this study, the purified feed AIN-93 M for 1 week (16.6 g/day/rat), the fecal weight of Group C showed no daily change (2.1/day/rat), suggesting that the intake of an MP-supplemented diet does not affect fecal weight. Hiroyuki et al. reported that when Wistar rats were fed a Western diet supplemented with 3% shrimp and crab shell powder, their fecal weight significantly increased compared with that of the control group^50^. Group K showed a significant and continuous increase in fecal weight from day 1 compared with that of Group C, confirming the phenomenon reported in previous studies. Matsuoka et al. reported that the addition of eggshell membrane to the AIN-76 diet in SD rats increased the weight of dry fecal material^51^. Similarly, in our experiment, the fecal weight in Group E increased significantly and consistently from day 2 compared with that in Group C, confirming the same phenomenon as that reported in previous studies. Wakabayashi et al. reported that feeding SD rats indigestible dextrin for 5 weeks (2 g/day/rat) increased fecal weight^34^. In contrast, Kato et al. reported that while cecal weight increased in Wistar rats fed indigestible dextrin for 21 days (0.9–1.1 g/day/rat), dry fecal weight did not^52^. In our study, the low intake of indigestible dextrin (0.8 g/day/rat) likely contributed to the absence of any effect, which is consistent with the results of Kato’s study. Additionally, Tanabe et al. reported no intergroup differences in fecal weight when a part of the AIN-93 M diet was replaced with isomalt oligosaccharides in Wistar rats, which is consistentwith our results^53^.

To analyze MPs in the fecal or gastrointestinal tract, 10% KOH is widely used to digest protein-rich materials owing to its hydrolysis in an alkaline environment. Kim et al. reported that under conditions of treatment in 10% KOH at 60℃ for 24 h, the recovery rates for fluorescent PE particles with diameters ranging from 125 to 150 μm were 88% ± 12% in rainbow trout fillets and 86% ± 10% in fish oil^43^. Karami et al. reported that treating eight types of MPs of < 300 μm and fish in 10% KOH at 40 °C for 24–48 h resulted in high recovery rates, with average recovery rates of 93.3–104.4%^42^. In this study, pre-treatment with 10% KOH resulted in recovery rates of > 95% for all the samples. Therefore, using 10% KOH for pre-treatment is considered appropriate for ensuring adequate decomposition of organic matter and achieving high recovery rates.

Experiments reported by Jang et al. showed that the amount of oil excreted in the stool of the experimental group was significantly higher than that in the control group after the addition of chitosan; moreover, high-molecular-weight chitosan formed gel aggregates with oil and bile salts in vitro^54^. This result is consistent with the promoting effect of chitosan on MP excretion observed in this study, supporting the hypothesis that chitosan enhances MP excretion by binding to other components of the gut. In addition, chitosan has been reported to dissolve in gastric juices and become a positively charged dietary fiber that binds to negatively charged bile acids in the gut^35^. A related study found that negatively charged ethyl cellulose can bind to a positively charged chitosan suspension in the small intestine of rats^55,56^. Some reports have suggested that chitosan may bind to its target substance through physical rather than chemical interactions^57^. Given the nonpolar nature of the polyethylene used in this study, the chemical effect of the charge is likely to be small. Therefore, it is necessary to further study the mechanism of action of chitosan, particularly the influence on different types and sizes of MPs.

The residual rate of MPs in the gastrointestinal tract of Group K was significantly lower than that in the other groups, implying that chitosan had the highest MP excretion effect among the candidate materials. This study suggests for the first time that chitosan binds with MPs in the gastrointestinal tract, facilitating their rapid excretion in fecal matter (MP fecal excretion rate: 115.6% ± 4.5%). Yoshimoto et al. reported that chitosan showed a high adsorption ability for nitrogen metabolites^58^ and that orally injected chitosan was excreted into feces by binding with cholesterol in the gastrointestinal tract^59^. Chitosan is assumed to absorb MPs and cholesterol, resulting in rapid excretion from the body. In contrast, for the MP assay method, the MP excretion rate in fecal exceeded 100% because the whole fecal sample could not be analyzed. Therefore, it is necessary to improve the accuracy of MP autolysis by increasing the sample volume and pre-treatment method.

The sum of the fecal MP excretion and retention rates in the gastrointestinal tract from 0 to 144 h was 95.8% in Group C, 104.2% in Group D, 101.9% in Group O, 121.7% in Group K, and 101.1% in Group E. These results suggest that MPs of 200 μm in size are unlikely to be absorbed into the body through the gastrointestinal tract and are mostly present in the gastrointestinal tract or excreted as fecal matter. Further research is needed as it is becoming clear that the prolonged presence of MPs in the gastrointestinal tract causes physical irritation to the gastrointestinal tract and affects the microbiota. For example, Lu et al. reported the effects of continuous ingestion of MPs on the intentional microbiota; polystyrene MP induced gut microbiota disorders and reduced mucus production in the colon^60^.

Lu et al. reported a significant decrease in T-cho and TG levels in mice compared with that of the non-MP intake group when mice were fed with PE MPs with an average particle size of 50 μm for 5 weeks (730 particles/day/mouse)^60^. Additionally, Deng et al. reported that when mice were administered fluorescent PS particles with a diameter of 20 μm for 4 weeks (2.27 × 10^4^ particles/day/mouse), T-cho and TG levels were significantly lower compared with those in the non-MP intake group^26^. In contrast, Kim et al. reported no significant changes in blood T-cho and TG levels in 8-week-old SD rats orally administered with PP particles with a diameter of 150 μm for 4 weeks (approximately 3700 particles/day/rat)^61^. Given that the intake of MPs in our study was approximately 3300 particles/day/rat, and the duration was only 7 days, it is reasonable to conclude that the intake of MPs over a short period is consistent with the findings of Kim et al. Further studies are needed to determine the extent to which MP intake affects blood marker levels.

Tissue weight measurements during dissection showed a significant increase in the gastrointestinal tracts of groups D and K compared with that in Group C. Wakabayashi et al. reported a significant increase in cecal weight after feeding 10% indigestible dextrin to 3-week SD rats for 5 weeks (34.4 g/day/rat)^62^. Yang reported a significant increase in colon weight after adding 10% chitosan to an AIN-93 M basal diet in 13-week SHRSP rats (13.7 g/day/rat)^63^. In this study, although the intake of indigestible dextrin and chitosan was lower than that in previous reports, a significant increase in gastrointestinal tract weight was observed, indicating that even over a short period of 7 days, an effect could be observed.

This study highlights two important aspects of the interactions between MPs and the digestive system. First, it shows that MPs may remain in the gastrointestinal tract even after a short period of ingestion; for ingestion of a basic diet, approximately 12% of ingested MPs remained in the gastrointestinal tract at 144 h post ingestion. Secondly, among the various indigestible substances, chitosan demonstrates a remarkable ability to promote the excretion of MPs. Chitosan is a natural polysaccharide derived from chitin and has been hypothesized to effectively bind to MPs and promote their elimination from the body. These findings suggest that chitosan is a valuable dietary supplement for reducing MP accumulation in the gastrointestinal tract.

## Conclusion

This study highlights two important aspects of the interactions between MPs and the digestive system. Firstly, it has been demonstrated that even short-term ingestion of MPs can result in their retention within the gastrointestinal tract. This finding suggests that MPs are not swiftly expelled from the body and can persist in the gastrointestinal system, potentially leading to cumulative exposure over time. The persistence of MPs, especially in the cecum, raises concerns about their potential long-term effects on gut health and overall physiological processes within the digestive system.

Secondly, among various indigestible materials tested in this study, chitosan showed a remarkable ability to facilitate the excretion of MPs. Chitosan, a natural polysaccharide derived from chitin, effectively binds MPs and promotes their removal from the body. The effectiveness of chitosan in promoting MP excretion suggests that chitosan is a valuable dietary supplement for reducing MP accumulation in the gastrointestinal tract.

## Electronic supplementary material

Below is the link to the electronic supplementary material.

Supplementary Material 1

## Data Availability

Data is provided within the manuscript or supplementary information files.
