# Supplementary material for: Ingesting chitosan can promote excretion of microplastics
Source: Sci Rep. 2025 Apr 23;15:14041. doi: 10.1038/s41598-025-96393-w (PMC12018927; doi:10.1038/s41598-025-96393-w)
Supplement: Supplementary file 1 — Supplementary Material 1 [file 41598_2025_96393_MOESM1_ESM.docx]

Ingesting chitosan can promote excretion of microplastics

Di Liu^1,2^, Muneshige Shimizu^1,2 *^

*^1^ Institute Oceanic Research and Development, Tokai University, 3-20-1 Orido, Shimizu, Shizuoka 424-8610, Japan*

*^2^ Graduate School of Science and Technology, Tokai University, 3-20-1 Orido, Shimizu, Shizuoka 424-8610, Japan*

Supplementary Table 1 provides detailed information on daily body weight of rats.

|  | day1 | day2 | day3 | day4 | day5 | day6 | day7 |
| --- | --- | --- | --- | --- | --- | --- | --- |
| group C | 310.32 | 314.35 | 319.45 | 321.92 | 326.48 | 329.80 | 329.83 |
| group D | 311.40 | 317.11 | 323.82 | 325.97 | 332.82 | 333.72 | 335.62 |
| group O | 311.12 | 315.51 | 320.41 | 322.95 | 326.47 | 329.87 | 329.72 |
| group K | 311.10 | 321.41 | 323.70 | 326.07 | 329.18 | 330.93 | 328.30 |
| group E | 307.55 | 310.96 | 315.45 | 315.98 | 319.92 | 321.15 | 321.78 |

Supplementary Table 2 provides detailed information on daily fecal weights of rats.

|  | day1 | day2 | day3 | day4 | day5 | day6 |
| --- | --- | --- | --- | --- | --- | --- |
| group C | 2.1 | 2.2 | 2.3 | 2.3 | 2.5 | 2.1 |
| group D | 2.0 | 2.5 | 3.1 | 3.1 | 3.0 | 2.9 |
| group O | 1.7 | 1.9 | 2.7 | 2.4 | 2.6 | 2.5 |
| group K | 3.4 | 4.9 | 5.1 | 4.7 | 5.1 | 5.0 |
| group E | 2.8 | 3.2 | 3.7 | 3.7 | 3.3 | 3.3 |

Supplementary Table 3 provides detailed information on the cumulative MP excretion rates in rats fecal.

|  | 0-24h | 0-48h | 0-72h | 0-96h | 0-120h | 0-144h |
| --- | --- | --- | --- | --- | --- | --- |
| group C | 14.8 | 35.5 | 59.5 | 72.4 | 78.4 | 83.7 |
| group D | 18.5 | 48.8 | 68.0 | 79.2 | 84.7 | 87.6 |
| group O | 19.8 | 40.2 | 60.0 | 72.1 | 80.3 | 86.2 |
| group K | 39.6 | 70.9 | 86.0 | 101.8 | 110.0 | 115.6 |
| group E | 27.2 | 47.4 | 67.7 | 81.4 | 84.2 | 90.6 |

Supplementary Table 4 provides detailed information on the MP excretion rate in the rat’s gastrointestinal tract

| group C | 12.1 |
| --- | --- |
| group D | 15.0 |
| group O | 13.4 |
| group K | 6.1 |
| group E | 10.5 |
